# A new target region for changing the substrate specificity of amine transaminases

**DOI:** 10.1038/srep10753

**Published:** 2015-06-01

**Authors:** Li-Jun Guan, Jun Ohtsuka, Masahiko Okai, Takuya Miyakawa, Tomoko Mase, Yuehua Zhi, Feng Hou, Noriyuki Ito, Akira Iwasaki, Yoshihiko Yasohara, Masaru Tanokura

**Affiliations:** 1 Department of Applied Biological Chemistry, Graduate School of Agricultural and Life Sciences, The University of Tokyo, 1-1-1 Yayoi, Bunkyo, Tokyo 113-8657, Japan; 2 Department of Ocean Sciences, Tokyo University of Marine Science and Technology, 4-5-7 Konan, Minato-ku, Tokyo 108-8477, Japan; 3 Biotechnology Development Laboratories, Kaneka Corporation, 1-8 Miyamae, Takasago, Hyogo 676-8688, Japan; 4 Research & Development Group, QOL Division, Kaneka Corporation, 1-8 Miyamae, Takasago, Hyogo 676-8688, Japan

## Abstract

(*R*)-stereospecific amine transaminases (*R*-ATAs) are important biocatalysts for the production of (*R*)-amine compounds in a strict stereospecific manner. An improved *R*-ATA, ATA-117-Rd11, was successfully engineered for the manufacture of sitagliptin, a widely used therapeutic agent for type-2 diabetes. The effects of the individual mutations, however, have not yet been demonstrated due to the lack of experimentally determined structural information. Here we describe three crystal structures of the first isolated *R*-ATA, its G136F mutant and engineered ATA-117-Rd11, which indicated that the mutation introduced into the 136^th^ residue altered the conformation of a loop next to the active site, resulting in a substrate-binding site with drastically modified volume, shape, and surface properties, to accommodate the large pro-sitagliptin ketone. Our findings provide a detailed explanation of the previously reported molecular engineering of ATA-117-Rd11 and propose that the loop near the active site is a new target for the rational design to change the substrate specificity of ATAs.

Pure chiral amines, especially the *R*-amines, are crucial building blocks in the synthesis of certain pharmaceutical drugs such as Cinacalcet used to treat secondary hyperparathyroidism^1^, an active ingredient Sitagliptin^2^ for diabetes mellitus type 2, Silodosin for the symptomatic treatment of benign prostatic hyperplasia^3^, Tamsulosin used in the treatment of difficult urination^4^, Formoterol for the management of asthma and chronic obstructive pulmonary disease^5^ and antihistamine Levocetirizine^6^. Amine transaminases (ATAs), trivially referred to as amine-pyruvate transaminases, have been a focus of increased attention due to their ability to synthesize pure chiral amines as promising biocatalysts. ATAs transfer an amino group of a chiral amine compound to a ketone compound using the cofactor pyridoxal 5’-phosphate (PLP) (Supplementary Fig. 1a) with a high turnover rate, stable catalytic activity, broad substrate specificity and excellent stereoselectivity^7^, which is achieved by a proposed large-binding pocket (L pocket) and small-binding pocket (S pocket) in the substrate-binding site^8^,^9^,^10^,^11^,^12^,^13^,^14^,^15^,^16^ (Supplementary Fig. 1b). This transfer ability could allow ATAs to overcome the shortcomings of chemical syntheses, such as harsh reaction conditions, effluent of toxic catalytic metal, and even insufficient enantiopurity^17^.

The first *R*-ATA, which was identified from *Arthrobacter* sp. KNK168 (FERM-BP-5228) (Ab-*R*-ATA) by enrichment screening, showed activity on primary amine and methyl ketones or small cyclic ketones^18^,^19^. Ab-*R*-ATA was successfully applied to the asymmetric synthesis of several kinds of chiral amines, such as (*R*)-dimethoxyamphetamine (DMA), (*R*)-4-methoxyamphetamine, (*R*)-1-(3-hydroxyphenyl) ethylamine and (*R*)-1-(3-methoxyphenyl) ethylamine^20^. A report on the purification, characterization, and gene cloning of Ab-*R*-ATA published recently^21^,^22^. Höhne *et al.* discovered 20 *R*-ATAs using an *in silico* search strategy based on the analysis of amino acid sequences of typical motif for *R*- and *S*-ATAs^23^. Some of the identified *R*-ATAs are under investigation on the substrate specificity and reaction conditions^24^. An improved *R*-ATA, designated as ATA-117-Rd11 was derived from ATA-117, a homolog of Ab-*R*-ATA, by the substrate walking approach for use in the synthesis of sitagliptin, on an industrial scale^25^,^26^, whereas both Ab-*R*-ATA and ATA-117 could not produce such a large compounds (Supplementary Fig. 1c). ATA-117-Rd11 achieved a 13% increase in yield and a 19% reduction in total waste, which ultimately reduced the production cost when compared to the chemical approach used to synthesize sitagliptin^9^. The series of studies on *R*-ATAs are widely regarded as a significant milestone in the history of enzyme application and engineering^27^,^28^,^29^. These breakthrough researches should be further investigated by experimental structural studies to more deeply elucidate the mechanism underlying the substrate specificity and/or stereospecificity of ATAs.

Up to now, several structures of *S*-ATAs^9^,^10^,^11^,^12^ and *R*-ATAs^13^,^14^,^15^,^16^ have been determined, providing some information about the dual substrate recognition^30^ of ATAs, which is a unique ability of transaminases to accept both the hydrophilic and hydrophobic substrates in the same active site. In the *S*-ATAs, a “flipping” arginine residue in a loop near the active site was proposed to play a key role in dual substrate recognition by moving the guanidino group in the active site to interact with the carboxylate of substrates or moving out of the active site to provide a hydrophobic environment^9^. Recently, *R*-ATAs from *Aspergillus terreus*, *Nectria haematococca* and *Aspergillus fumigatus* were reported and an arginine residue near the active site was assumed to possess the similar function, binding carboxylated groups, as that in the *S*-ATAs^14^,^15^,^16^.

Although several structures of *S*-ATAs and *R*-ATAs are already available, the structural basis for the substrate specificity and enantioselectivity of the engineered ATA-117-Rd11^25^,^26^ has not been elucidated yet. The molecular engineering of ATA-117-Rd11 should be reevaluated by structural study of the enzyme because, once a protein engineering is rationalized by experimental structural information, the extensive data gathered during the engineering process and the essence extracted from the data may be widely applied to some other enzymes and will drive researches on enzyme engineering. Here we show three crystal structures of the wild-type Ab-*R*-ATA, its G136F mutant, and ATA-117-Rd11, along with mutational and computational analysis. In this study, we used Ab-*R*-ATA instead of ATA-117, the parental enzyme of ATA-117-Rd11, and could elucidate a reasonable molecular mechanism underlying the engineering of ATA-117-Rd11 because the difference between Ab-*R*-ATA and ATA-117 is only the amino acid residue at position 306 (Val and Ile in Ab-*R*-ATA and ATA-117, respectively) far from the active site, which is supposed to have negligibly small effect on the substrate recognition and activity and is not conserved in *R*-ATAs. In addition, the 306^th^ residue and the residues around it were not included in any round of evolution during the engineering. The G136F mutant was derived from structural comparison between Ab-*R*-ATA and ATA-117-Rd11, where the 136^th^ residue was considered as the main cause of the structural alteration of the loop near the active site as will be described. These results would provide the molecular basis for the conversion of the substrate specificity of ATAs, which are highly valuable biocatalysts.

## Results

### Overall structures

**Figure 1:**
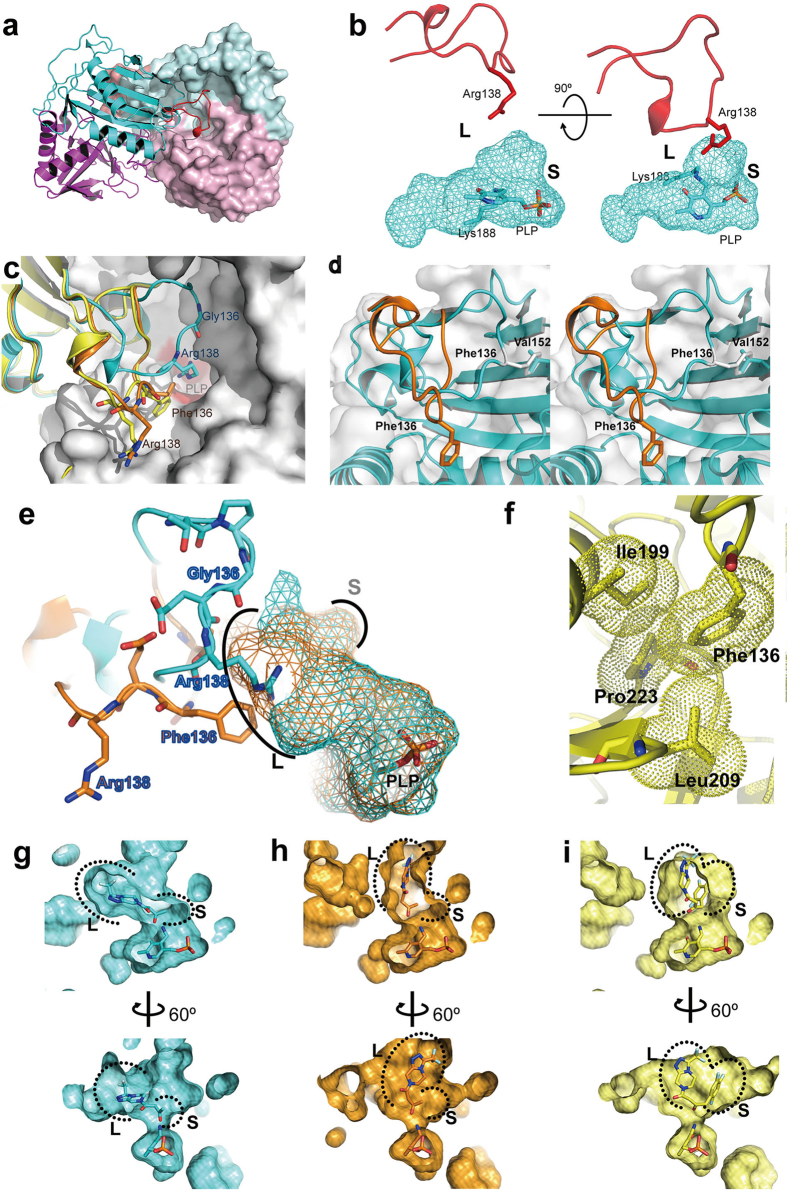
Structural architecture of R-ATAs and structural conversions in the loop 129-145. (**a**) The Ab-*R*-ATA dimer. A protomer is shown as a ribbon and the other as the surface. The large and small domains are colored in cyan and magenta, respectively, except for the loop 129–145, which is colored in red. (**b**) The L pocket and S pocket in the active site. The cyan mesh represents the cavity of the active site. PLP and the side chain of Lys188 are shown as sticks. The loop 129–145 and Arg138 from the neighboring protomer are shown as a red ribbon and stick, respectively. (**c**) Structural comparison among the loops 129–145. The backbone trace around the loops 129–145 of Ab-*R*-ATA, the mutant G136F, and ATA-117-Rd11 are shown in cyan, orange, and yellow, respectively. The residues 136 and 138 and PLP are shown as sticks. The neighboring protomer of Ab-*R*-ATA is shown as a white surface with a red substrate-binding site. (**d**) Stereo view showing the probable conformational change of loop 129–145. A steric hindrance occurred between Phe136 and Val152 once Gly136 of Ab-*R*-ATA (cyan) was manually mutated to phenylalanine (white) in its energy-favorable rotamer. To avoid this steric hindrance, the loop changes its conformation to that of the G136F mutant (orange). (**e**) Enlargement of the L pocket caused by the mutation of G136F. The cavity of Ab-*R*-ATA and the G136F mutant are shown as cyan and orange mesh, respectively. (**f**) Close-up view of the residue Phe136 in ATA-117-Rd11. The van der Waals radii of the side chains of Phe136, Ile199, Leu209 and Pro223 are represented as yellow dots. (**g** to **i**) Changed position of the L and S pockets. The docking models of the methyl ketone analog docked into Ab-*R*-ATA (**g**) or the G136F mutant (**h**), and pro-sitagliptin ketone docked into the ATA-117-Rd11 (**i**) are shown. The parameters for cavity-detection and presentation are consistent among these three panels.

The crystal structures of the wild-type Ab-*R*-ATA, the G136F mutant of Ab-*R*-ATA and ATA-117-Rd11 were determined at 1.65-Å, 2.27-Å, and 2.20-Å resolutions, respectively (Supplementary Table 1). These structures are very similar to each other (RMSD of Cα atoms less than 0.38 Å). Each protomer (subunit) of the dimeric enzymes shows a structural architecture typical of the fold-type IV class of PLP-dependent enzymes, which are made up of two domains (Fig. 1a and Supplementary Fig. 2). The substrate-binding site is surrounded by the cavity among the two domains, the cofactor PLP (Supplementary Fig. 3), and the loop consisting of the residues 129–145 of the other protomer (Fig. 1a). The substrate-binding site can be divided into two parts as described initially, the L pocket and the S pocket (Fig. 1b), which is consistent with the previously proposed mechanism for the substrate specificity of ATAs in which the smaller and larger atomic groups of substrates are recognized by the S and L pockets, respectively^8^. The results of docking simulations using these crystal structures supported the mechanisms (Supplementary Fig. 4).

### Arg138 in the loop 129-145 of Ab-*R*-ATA concerns dual substrate recognition

The models complexed with a glycerol molecule at the active site of Ab-*R*-ATA (Supplementary Fig. 5) suggested that Arg138 of the wild-type Ab-*R*-ATA is important for the recognition of probable native substrates, such as pyruvate. This suggestion was supported by site-directed mutagenesis analyses. The *k*_cat_/*K*_m_ values for pyruvate of the R138A and the R138Q mutants, using (*R*)-1-phenylethylamine as the amino donor, were about 350-fold and 500-fold lower than that of the wild-type Ab-*R*-ATA, respectively, indicating that the side chain of Arg138 is important for the recognition of pyruvate (Supplementary Table 2). The importance of Arg138 in the pyruvate recognition was also supported by molecular dynamics (MD) simulations of the wild-type Ab-*R*-ATA complexed with PMP-pyruvate quinonoid intermediate. During the simulation, the carboxyl group of the quinonoid intermediate formed a stable salt bridge with guanidinium group of Arg138 (Supplementary Fig. 6).

Ab-*R*-ATA can also transfer amino group to the ketones with highly hydrophobic larger substituents (R_L_), such as benzylacetone. The specific activity of the R138A and the R138Q mutants towards benzylacetone, using (*R*)-1-phenylethylamine as the amino donor, decreased to 50% and 23%, respectively, as the results of the mutations (Supplementary Table 2), indicating that the Arg138 residue plays an important role when Ab-*R*-ATA forms the complex with benzylacetone, although the details remained to be established.

### Significantly different conformations in the loop 129-145

The loops 129–145 of the G136F mutant and ATA-117-Rd11 showed significantly different conformation from that of the wild-type, depending on the amino acid residue at the position 136 (Fig. 1c, Supplementary Figs. 7-8). Gly136 was located near Val152 in the wild-type, whereas Phe136 was distant from Val152 in the G136F mutant and ATA-117-Rd11, accompanied by the long-range alteration of the loop’s backbone conformation (Fig. 1d). This suggests that the probable steric hindrance between the bulky side chain of Phe136 and Val152 was the main cause of the conformational difference. The G136F/Y/H/W mutants show more than 40-fold lower *k*_cat_/*K*_m_ values towards pyruvate (Supplementary Table 2), indicating that these mutants could not recognize pyruvate properly. This is probably due to the introduction of a bulky side chain into the residue 136, which could have altered the conformation of the loop 129–145, and thus caused Arg138, the key residue for pyruvate recognition, to move out from the active site (Fig. 1c).

### Structural conversions located beyond the loop 129-145

**Figure 2:**
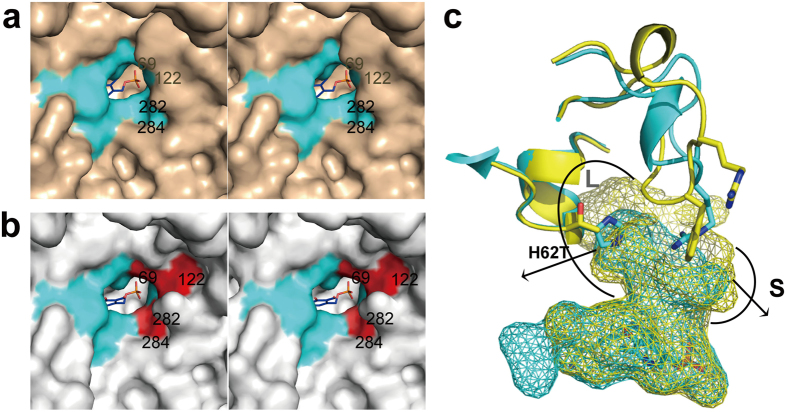
Structural conversions located beyond the loop in the molecular engineering to produce sitagliptin. (**a** and **b**) Enlargement of the S pocket caused by the four mutations in the active sites. Stereo view of the surface is shown for Ab-*R*-ATA (**a**) and ATA-117-Rd11 (**b**) The locations of the four residues are shown in red and labeled. (**c**) Enlargement of the L pocket (semi-circle labeled L) caused by the mutation H62T. His62 (in the L pocket of Ab-*R*-ATA) was mutated to Thr, reducing its size and increasing the volume of the active site around it. The active site cavities of Ab-*R*-ATA (cyan) and ATA-117-Rd11 (yellow) are shown as mesh. The semi-circle-labeled S represents the enlarged S pocket mentioned in (**b**).

Cumulative mutations on the substrate-binding site served to enlarge its volume and change its surface properties. Based on the results of activity detection of the engineering study, the truncation of Phe122 and either Val69 or Ala284 of ATA-117 is adequate for accommodating the 1,2,4-3-fluorine phenyl group of pro-sitagliptin ketone^25^,^26^. In the final biocatalyst ATA-117-Rd11, Val69, Phe122, and Ala284 (in the S pocket of Ab-*R*-ATA) were mutated to Thr, Met, and Gly, respectively. Comparison of the active site pockets of the Ab-*R*-ATA and ATA-117-Rd11 shows that the volume of S pocket is enlarged by the three mutations (Fig. 2a–b). The mutation of V69T, which does not seem to contribute to the change in the volume of the substrate-binding site, may contribute to the stability of the enzyme, since mutations including the 69^th^ residue allowed the enzyme to react under more harsh conditions^25^. Similarly, His62 (in the L pocket of Ab-*R*-ATA) was mutated to Thr to increase the volume of the active site around it by reducing the volume of the amino acid side chain (Fig. 2c).

## Discussion

Dual substrate recognition is a characteristic of ATAs. Both the hydrophilic and hydrophobic groups of substrates can be bound to the same L pocket of ATAs. For *S*-ATAs, as mentioned in the introduction, the dual substrate recognition is achieved via the conformational change of a “flipping” arginine residue in a loop near the active site^9^. For *R*-ATAs, similarly, an arginine residue near the active site was supposed to show the similar function to that in the *S*-ATAs^14^,^15^,^16^; Arg126 in the *R*-ATA from *Nectria haematococca* or *Aspergillus fumigatus* and the Arg128 in the *R*-ATA from *Aspergillus terreus*. Although the corresponding arginine residue is not conserved in Ab-*R*-ATA (Supplementary Fig. 9), the guanidinium group of Arg138 of Ab-*R*-ATA was located near the position where the guanidinium groups of those arginine residues in the other *R*-ATAs (Supplementary Fig. 10). In addition to this structural similarity, Arg138 of Ab-*R*-ATA was proven to recognize the carboxyl group of pyruvate by kinetic analysis and MD simulation, suggesting that Arg138 is the compensation for the key arginine residue of the other ATAs.

However, in contrast to that in the *S*-ATAs, the corresponding arginine residues in the *R*-ATAs did not show any flipped conformation. Instead, two conformations of an active site loop (corresponding to the loop of Arg129-Pro145 of Ab-*R*-ATA) containing the notable arginine residue in the *R*-ATA from *Aspergillus fumigatus* were observed^14^. In the “open” conformation, the active site loop moved out of the active site and stabilized by the hydrogen bond between Arg126 and Asp132 residues, which probably could provide more space and stronger hydrophobic environment to accommodate a bulky hydrophobic substrate. Although a similar loop conformation was not observed in the structure of Ab-*R*-ATA, it can be supposed to exist during the reaction, stabilized by the interaction between Arg138 and some negative charged side chains or the carbonyl group of main chain. This hypothesis can explained our results of activity measurements of the R138A and the R138Q mutants toward benzylacetone. When Arg138 was mutated to alanine or glutamine, the interaction that could maintain the loop 129–145 in an “open” conformation became weak so that the loop may adopt the conformation in the crystal structure, which is unfavorable for benzylacetone binding. Thus, the specific activities of the R138A and the R138Q mutants towards benzylacetone were decreased. It is noticeable that, from the discussion above, the dual substrate recognition mechanism of *R*-ATAs probably differs from that of *S*-ATAs. *R*-ATAs alter the conformation of the active site loop containing an arginine residue, while *S*-ATAs flip a single arginine residue.

**Figure 3:**
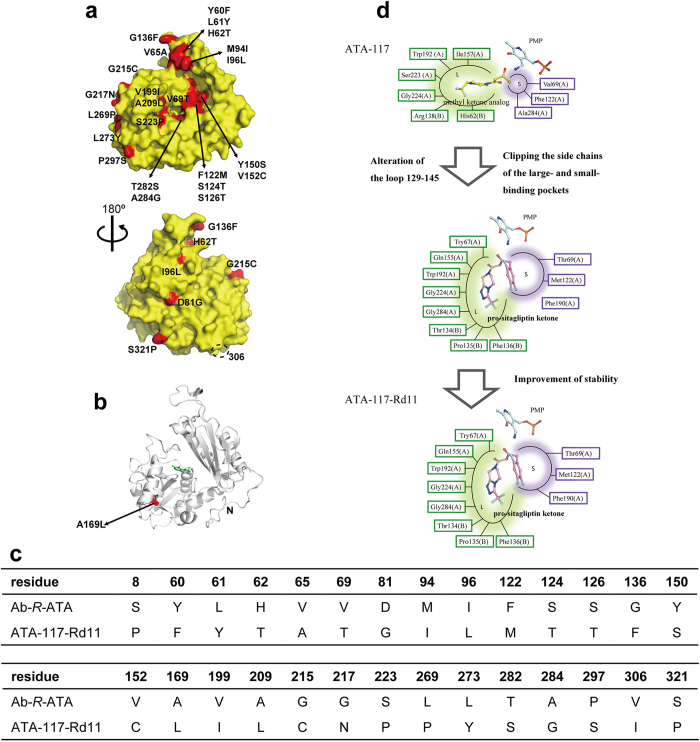
Analysis of the previous molecular engineering of ATA-117. (**a**-**c**) Amino acid sequence comparison among Ab-*R*-ATA, ATA-117, and ATA-117-Rd11. The 27 mutations introduced in the previous successful engineering of ATA-117 on the molecular surface (**a**) or inside the molecule (**b**) are highlighted in red and labeled. Residue 306 is indicated by the dashed circle in (**a**) .The “N” in (**b**) represents the Val11 at the N-terminus. The unobserved Phe8 in ATA-117-Rd11 is probably away from the active site. The cofactor PLP in the active site is shown as green sticks. The table in (**c**) shows the differences of the amino acid residues among three *R*-ATAs. (**d**) A schematic diagram of the strategies in the engineering. The amino acid residues consisting of the L pocket and S pocket are represented in green and purple text boxes, respectively. Cofactor PMP and docked ligands are shown as sticks.

The molecular engineering of ATA-117-Rd11 involved 27 mutations (Fig. 3a–c) that made this ATA conquer the severe reaction problems in the realistic industrial production, such as a high concentration of organic solvent, a high temperature for enhancing the solubility of prositagliptin ketone, and a high concentration of amine donor isopropylamine for prompting the product accumulation^25^,^26^. Another notable point is that the high stereoselectivity was not lowered by the 27 mutations^25^,^26^. Based on the previous engineering study and the crystal structures in the present study, the 27 mutations can be divided into three kinds of structural modifications: 1) alteration of the conformation of the loop 129–145, 2) clipping or replacement of the side chains of the residues composing the substrate-binding site (including the L or S pockets), and 3) modifications of residues out of the substrate binding pockets for improving biochemical characteristics (Fig. 3d). Among these three kinds of modifications, the first and second can be explained by our crystal structures.

As described above, the alteration of the loop 129–145 varied the properties of the substrate binding site drastically. The residue 136 was moved into the L pocket by the alteration of the loop, leading to a probable hydrophobic interaction between Phe136 and the cyclic group of pro-sitagliptin ketone (Fig. 3d). Simultaneously, Arg138 was moved away from the substrate-binding site by the alteration of the loop, resulting in an enlargement of the cavity to accommodate large substrates (Fig. 1e). These conclusions could well explain how the G136F/Y/H/W mutants favor bulky and hydrophobic substrates. In addition, along with the other structural modifications, the position of the L pocket shifted (Fig. 1g–i). Ser223 (in the L pocket of Ab-*R*-ATA) was mutated to Pro, resulting in an additional interaction, a van der Waals interaction among the side chains of Phe136, Ile199, Leu209, and Pro223, which would have stabilized the conformation of the loop 129–145 (Fig. 1f). To sum up these mutational effects, the volume of the substrate-binding site was drastically enlarged by the alteration of the loop 129–145 and the other mutations, and the position of the L pocket was shifted by the alteration of the loop 129–145. Based on these results, we propose that the loop 129–145 mainly determines the substrate specificity of Ab-*R*-ATA, although cumulative mutations on the substrate-binding site also serve to enlarge its volume and change its surface properties (Fig. 2).

**Figure 4:**
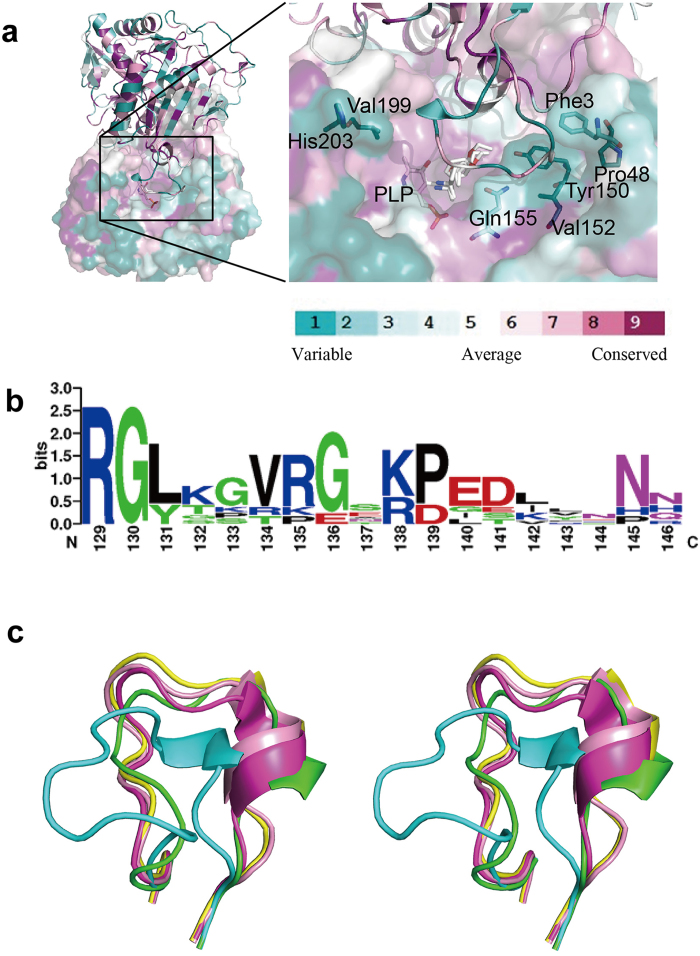
Conservation of the residues in the active site and loop 129-145. (**a**) The amino acid residues on the molecular surface of Ab-*R*-ATA are colored turquoise to maroon according to the conservation grades shown on the color-coding bar, which range from variable to conservative. The conservativeness was evaluated by analyzing the aligned sequence of Ab-R-ATA and its seven homologs on the ConSurf server^41^,^42^. The seven homologs of Ab-*R*-ATA are from *Aspergillus terreus* (GI_115385557), *Penicillium chrysogenum* (GI_211591081), *Aspergillus oryzae* (GI_169768191), *Aspergillus fumigatus* (GI_70986662), *Neosartorya fischeri* (GI_119483224), *Gibberella zeae* (GI_46109768), *Mycobacterium vanbaalenii* (GI_120405468). (**b**) Logographical representations of the amino acid sequence conservation within loop 129–145 in Ab-*R*-ATA and the seven homologs. The height of each stack corresponds to the degree of sequence conservation at that position. The height of letters represents the relative frequency of individual amino acids at the position. Logo representations were obtained by using Weblogo. (**c**) *R*-ATAs showed different conformations of the corresponding loop 129–145. These loops of Ab-*R*-ATA, ATA-117-Rd11, *R*-ATA from *Aspergillus terreus* (PDB ID 4CE5), *R*-ATA from *Aspergillus fumigatus* (PDB ID 4CHI) and *R*-ATA from *Nectria haematococca* (PDB ID 4CMD) are shown in cyan, green, magenta, yellow and pink, respectively.

From the alignment of Ab-*R*-ATA and eight *R*-ATAs recently identified^13^,^23^, most of the residues that make up the substrate-binding site of Ab-*R*-ATA are conserved in the other seven enzymes, whereas the residues in the loop 129–145 are not conservative (Fig. 4a–b and Supplementary Fig. 9). The difference among the substrate specificities of the seven newly discovered *R*-ATAs is discussed based on the phylogenetic relationship among them, and *R*-ATAs from *Aspergillus terreus* and *Penicillium chrysogenum* showed distinct specificity although they have high similarity score in multiple alignments^24^. There is only one different residue in the loop between them. Meanwhile, another two *R*-ATAs from *Aspergillus fumigatus* and *Neosartorya fischeri* with the same sequence in the loop showed nearly the same substrate specificity^24^. Thus, it is reasonable to assume that the diversity of the amino acid sequence of the loop causes the different substrate specificity among these enzymes, in accordance with the present model, in which the loop 129–145 is the main determinant of the substrate specificity of *R*-ATA. The recently determined crystal structures of *R*-ATAs^13^,^14^,^15^,^16^ show different conformations of the corresponding loop (Fig. 4c) from that of Ab-*R*-ATA and ATA-117-11Rd, supporting our suggestion that the loop’s conformation can vary depending on the amino acid sequence. In addition, mutation of the residues Phe3, Pro48, Tyr150, Gln155, Val199, and His203, which exist around the loop 129–145 (Fig. 4a), are also expected to change the conformation of this loop. As a conclusion, the loop 129–145 functions as a substrate-specificity determinant, and this loop and its surrounding residues should be prioritized as research subjects when attempting to engineer *R*-ATAs to transform the substrate specificity.

## Methods

### Overexpression and purification

The Ab-*R*-ATA expression plasmid constructed by inserting the *R*-ATA gene from *Arthrobacter* sp. KNK168 (FERM-BP-5228) (GI_ 336088340) under the *lac* promoter of the pUCNT vector^22^ was used for transformation of *Escherichia coli* Rosetta (DE3) to overexpress Ab-*R*-ATA that contains 330 amino acids with no tag. The transformants were cultivated at 37 °C in Lysogeny broth (LB) medium containing 100 μg/mL ampicillin. Overexpression was induced by adding 1 mM isopropyl-β-D-thiogalactopyranoside (IPTG) when the optical density at 600 nm reached 0.6, and the culture was continued 20 hours at 20 °C. The expression plasmid for the G136F mutant of Ab-*R*-ATA was constructed using a PrimeSTAR Mutagenesis Basal Kit (Takara, Shiga, Japan). The DNA fragment containing the gene of ATA-117-Rd11 was synthesized (GenScript USA Inc.). The ATA-117-Rd11 gene was amplified with KOD-plus DNA polymerase (Toyobo, Tokyo, Japan) and inserted into the *Nde*I-*Sac*I site of pET28a (+) to overexpress the fusion protein with an N-His tag. The G136F mutant and ATA-117-Rd11 were expressed in the same way as Ab-*R*-ATA.

To purify Ab-*R*-ATA and the G136F mutant, the cells were resuspended in lysis buffer containing 20 mM potassium phosphate buffer (pH 6.8), 0.01% 2-mercaptoethanol, 1 mM PLP and 10% (v/v) glycerol. The cells were disrupted by sonication and centrifuged at 40000 *g* at 4 °C for 30 min. The supernatant was applied to a DEAE-Sepharose column, and the protein was eluted with 400 mM NaCl dissolved in buffer A containing 20 mM potassium phosphate buffer (pH 6.8) containing 1 mM dithiothreitol (DTT) and 10% (v/v) glycerol. The eluted protein with 1 mM PLP additive was dialyzed against buffer A. The dialyzate was applied to a 6 mL ResourceQ column (GE Healthcare) equilibrated with buffer A, and the protein was eluted with an NaCl gradient ranging from 0 to 1 M. The purified fractions were concentrated and loaded on an equilibrated Superdex 200 HR 10/30 column (GE Healthcare) and eluted with 20 mM potassium phosphate buffer (pH 6.8) containing 1 mM DTT and 5% (v/v) glycerol. The purified fractions were concentrated to 8 mg/mL using a Vivaspin-20 concentrator (10000 molecular weight cutoff). To purify ATA-117-Rd11, the supernatant after disrupting and centrifuging was applied to a Ni-Sepharose column, and ATA-117-Rd11 was eluted with 200 mM imidazole dissolved in buffer A. Before dialyzing against buffer A, 1 mM PLP and 500 U thrombin protease (GE Healthcare) were added into the eluted protein. The dialyzed protein was applied to a Ni-Sepharose column once again to remove the uncleaved protein. Subsequent ion exchange chromatography and size exclusion chromatography were performed as described above.

### Crystallization

All crystallization experiments were performed at 20 °C by the sitting-drop vapor-diffusion method. The crystallization drops were prepared by mixing 2.0 μL of protein solution and 2.0 μL reservoir solution. After the crystallization conditions had been refined, crystals of Ab-*R*-ATA suitable for X-ray analysis were obtained using a reservoir solution consisting of 0.2 M magnesium chloride, 0.1 M Bis-Tris (pH 6.4), and 22% (w/v) PEG 3350. Before crystallization of ATA-117-Rd11, the protein storage buffer was changed to 20 mM Bis-Tris buffer (pH 6.8) containing 1 mM DTT and 10% (v/v) glycerol. By streak seeding, the crystals grew in one week. The crystallization drops were prepared by mixing 1.5 μL protein solution (15 mg/mL) and 1.5 μL reservoir solution (0.2 M magnesium chloride, 0.1 M Tris-HCl (pH 8.3), 18% PEG 3350). Crystals of the G136F mutant of Ab-*R*-ATA grew in a drop containing 1.0 μL protein solution (10 mg/mL) and 1.0 μL reservoir solution (0.2 M magnesium chloride, 0.1 M HEPES-HCl (pH 7.8), 16% PEG 3350) in 5 days.

### Data collection and processing

The presence of glycerol and PEG 3350 under the crystallization condition alleviated the need for subsequent cryoprotection, and thus crystals were picked up in a nylon loop (Hampton Research) and directly flash-cooled in a nitrogen cryostream (100 K). The data sets for Ab-*R*-ATA, ATA-117-Rd11 and the G136F mutant were collected at beamlines BL-5A, AR-NW12A and BL-5A at the Photon Factory (Tsukuba, Japan), respectively. All data sets were composed of 360 images and collected using a 0.5° oscillation. All the diffraction data were indexed, integrated and scaled with XDSme^31^. Data-collection statistics are given in Supplementary Table 1.

### Structure determination and refinement

The initial phasing of Ab-*R*-ATA diffraction data was conducted by molecular replacement using MOLREP^32^ in the CCP4^33^ and with a single molecule of branched-chain amino acid aminotransferase as the search model (PDB 1IYE). The initial solution with one molecule in the asymmetric unit had starting *R*_work_ and *R*_free_ values in excess of 50%, which only dropped slightly, respectively, after refinement with Refmac5^34^. The model was therefore improved using Buccaneer^35^ and ARP/wARP^36^, and these software successfully built most of the structure. The structure was then refined using Refmac5 and Phenix^37^ to obtain a model containing water with *R*_work_ and *R*_free_ values of 18.0% and 22.7%, respectively. The structure refinement was completed in Coot^38^ and Refmac5. The crystal structures of ATA-117-Rd11 and the G136F mutant were solved by molecular replacement using the structure of the Ab-*R*-ATA as a search model. The structures were manually rebuilt using Coot and refined using Refmac5.

### Molecular docking

The docking simulations were performed using the software package MOE2011.10 (Chemical Computing Group, Montreal, Canada). The dimers of the crystal structures of Ab-*R*-ATA, ATA-117-Rd11 and the G136F mutant with some modifications (the internal aldimines were changed to lysine and PMP) were used as the receptor model. The missing hydrogen atoms of these dimers were generated and the energy was minimized using the Merck molecular force field 94x (MMFF94x) with distance-dependent dielectric electrostatics. The possible conformations of the methyl ketone analog and pro-sitagliptin ketone were generated using the systematic mode and used for the simulation. The two ligands were docked to each of the receptor models on the possible active sites, which were identified by the function Site Finder, using an Induced Fit protocol with default values for all parameters. The scoring function used by the Dock module of MOE is based on protein-ligand interaction energies. Solutions that met the following criteria were selected from the results of the Dock module of MOE and applied to the analysis: 1) the reactive keto group of the ligand points towards the -NH_2_ of PMP within an appropriate distance (less than 4.0 Å), and 2) the interaction energy and binding scores are favorable.

### Molecular dynamics simulations

The model of the wild-type Ab-*R*-ATA complexed with PMP-pyruvate quinonoid intermediate was generated by using software MOE2014.09 (Chemical Computing Group, Montreal, Canada) from the atomic coordinates of the wild-type Ab-*R*-ATA complexed with PLP in the internal aldimine form. The Schiff linkage of the internal aldimine was removed and the quinonoid intermediate was built by adding atoms to the PLP residue. The quinonoid and the side chain of Lys188 were energy-minimized using AMBER12:EHT force field with distance-dependent dielectric electrostatics, while the other atoms are fixed. The homodimer of Ab-*R*-ATA was generated by using crystallographic symmetry operation.

Preparation of system was also carried out with MOE2014.09. The missing hydrogen atoms were generated by Protonate 3D function and energy-minimized using AMBER12:EHT force field with distance-dependent dielectric electrostatics. Sodium ions for neutralization and explicit water molecules were placed around the protein and the cubic periodic boundary was set at least 4-Å apart from the protein surface. The ions and solvent molecules were energy-minimized and applied to 450-ps dynamics simulation in order to be fit to the protein surface appropriately. All atoms in the system were then energy-minimized. Gradually decreased tether weight was applied to all non-hydrogen atoms during this final minimization steps.

Molecular dynamics simulation was carried out using software NAMD 2.10^39^ using the input file generated by MOE2014.09. The first 1-ns simulation, which was not used for trajectory analysis, was performed with the gradually decreasing positional restrains for non-hydrogen atoms. Then, molecular dynamics simulation without restrains were performed and used for trajectory analysis. All the dynamics simulations using NAMD were carried out with the time step of 1 fs in NVT ensemble at pressure of 101 kPa and at temperature of 300 K without any bonding constraints. Langevin method was used to control the constant temperature and pressure. The short range interactions were cut off at 10 Å for van der Waals and electrostatic interactions and long-range electrostatic interactions were calculated using the particle-mesh Ewald method. Obtained trajectory was analyzed by using software VMD^40^.

### Enzyme assay and analysis

Transaminase activity was assayed by measuring the production of acetophenone, as described previously with minor modifications^22^. Briefly, the reaction mixture contained 10 mM (*R*)-1-phenylethylamine, 10 mM pyruvate or benzylacetone, 100 mM Tris-HCl (pH 8.5), 0.5 mM PLP and the enzyme solution. The reaction mixture was incubated at 30 °C for 60 min and stopped by adding HCl. The amount of acetophenone formed was determined by HPLC. For determination of kinetic parameters, reaction mixtures containing 0.625–2000 mM pyruvate were used. Before initializing the reaction by the addition of enzyme, the pH of the reaction solution was adjusted when the pyruvate concentration is high. The reaction time was adjusted to 30 min.

## Additional Information

**How to cite this article**: Guan, L.-J. *et al.* A new target region for changing the substrate specificity of amine transaminases. *Sci. Rep.* **5**, 10753; doi: 10.1038/srep10753 (2015).

## Supplementary Material

Supplementary Information
