# Supplementary material for: A new target region for changing the substrate specificity of amine transaminases
Source: Sci Rep. 2015 Jun 1;5:10753. doi: 10.1038/srep10753 (PMC5377235; doi:10.1038/srep10753)
Supplement: Supplementary Information [file srep10753-s1.pdf]

Supplementary Materials for

A new target region for changing the substrate specificity of amine transaminases

Li-Jun Guan[1,+], Jun Ohtsuka[1,+], Masahiko Okai[1,2], Takuya Miyakawa[1], Tomoko Mase[1], Yuehua Zhi[1], Feng Hou[1], Noriyuki Ito[3], Akira Iwasaki[4], Yoshihiko Yasohara[3], Masaru Tanokura[1,*]

[1]Department of Applied Biological Chemistry, Graduate School of Agricultural and Life Sciences, The University of Tokyo, 1-1-1 Yayoi, Bunkyo, Tokyo 113-8657, Japan

[2]Department of Ocean Sciences, Tokyo University of Marine Science and Technology, 4-5-7, Konan, Minato-ku, Tokyo 108-8477, Japan

[3]Frontier Biochemical & Medical Research Laboratories, Kaneka Corporation, 1-8, Miyamae, Takasago, Hyogo 676-8688, Japan

[4]Research & Development Group, QOL Division, Kaneka Corporation, 1-8 Miyamae, Takasago, Hyogo 676-8688, Japan

[5]These authors contributed equally to this work.

*Corresponding author. E-mail: amtanok@mail.ecc.u-tokyo.ac.jp, Tel: 03-5841-5165, Fax: 03-5841-8023

This PDF file includes:

Supplementary Figures 1-10

Supplementary Tables 1-2

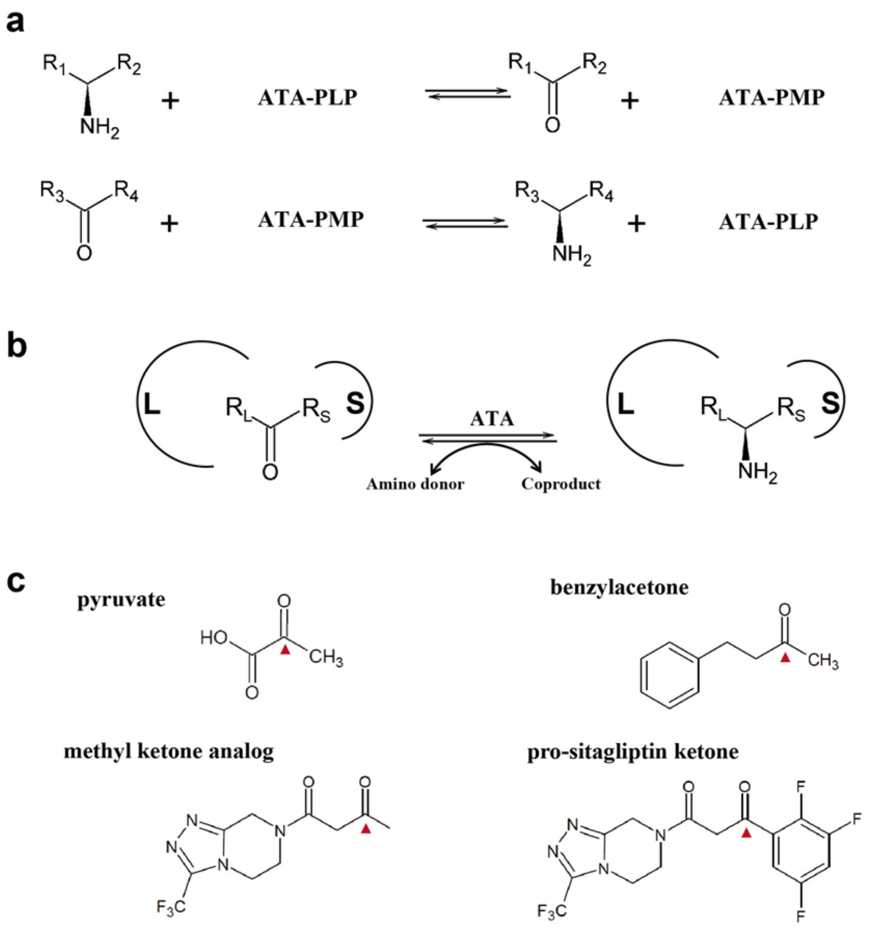

Supplementary Figure 1. The reaction system of amine transaminases (ATAs). (**a**) Two half reactions comprising the transaminase reaction. (**b**) Two binding-site model with a large-binding pocket (L) and a small-binding pocket (S). R_S and R_L represent the small sized alkyl group and large sized alkyl/aryl group, respectively. (**c**) The structures of the organic compounds pyruvate, benzylacetone, methyl ketone analog and pro-sitagliptin ketone used in the reaction system of Ab-*R*-ATA or ATA-117-Rd11. The red arrow heads represent the reactive carbonyl group.

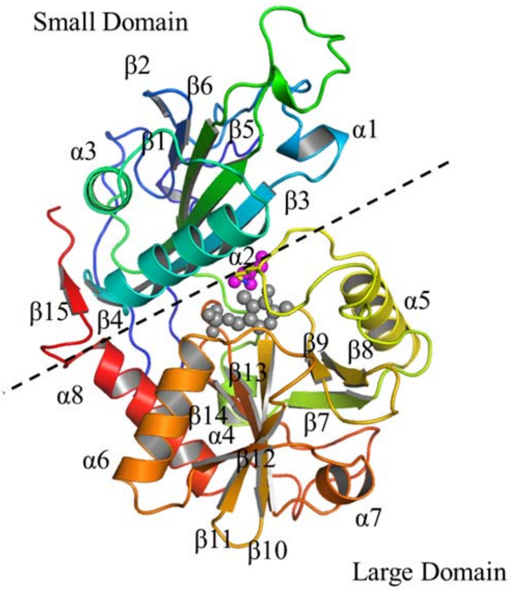

Supplementary Figure 2. Overall structure of Ab-*R*-ATA. The Ab-*R*-ATA protomer is colored in a rainbow representation from the N-terminus in blue to the C-terminus in red. Secondary structure elements are labeled. PLP (grey) and glycerol (magenta) between the large domain and small domain are shown as spheres. The glycerol molecule came from the protein solution.

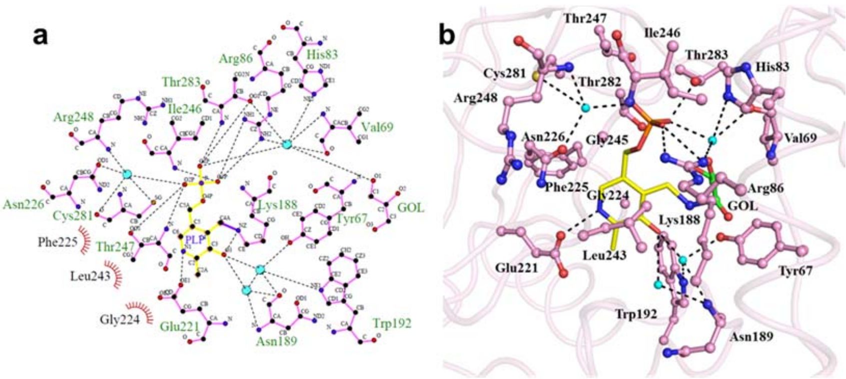

Supplementary Figure 3. Detailed structural representation of the interactions between PLP and Ab-*R*-ATA. (**a**) LIGPLOT representation of the PLP and Ab-*R*-ATA. Hydrophilic interacting atoms are connected by black dashed lines; nonligand residues involved in direct hydrophobic contacts with PLP are shown as red semicircles with radiating spokes. The Schiff base between the PLP and Lys188 residue of Ab-*R*-ATA are shown in purple. Water molecules are shown as cyan circles. (**b**) Three-dimensional residue interaction map illustrating the amino acids within 4 Å from PLP. PLP is shown as yellow sticks. Residues within the binding pocket are shown as a ball-and-stick representation in pink.

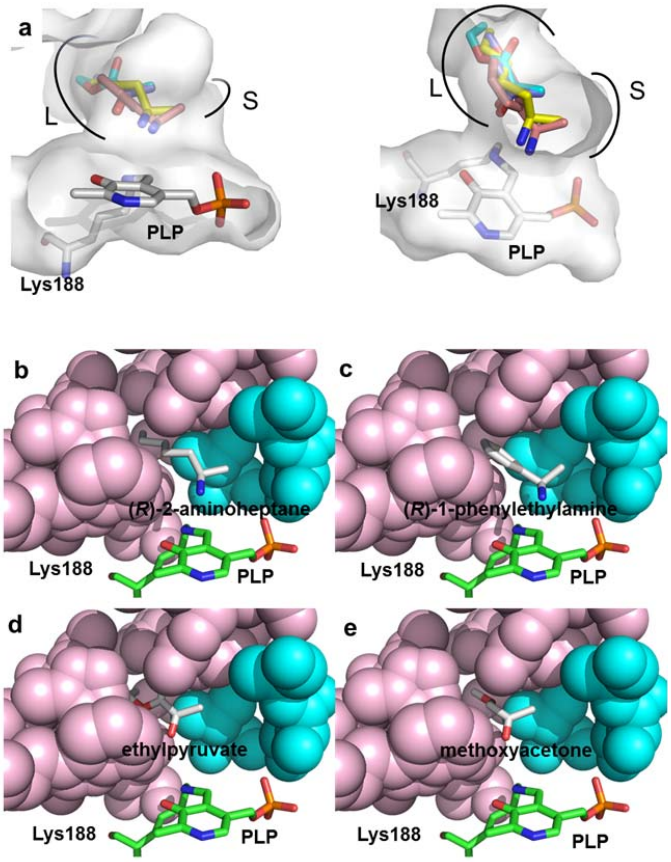

Supplementary Figure 4. The large binding pocket and small binding pocket of Ab-*R*-ATA. (**a**) The active site cavity (white surface) and the docking models of Ab-*R*-ATA and (*R*)-2-aminoheptane (yellow), (*R*)-1-phenylethylamine (orange), ethylpyruvate (cyan) and methoxyacetone (magenta). The internal aldimine formed by PLP and Lys188 is shown as white sticks. (**b**), (**c**), (**d**), and (**e**) are close-up views of the binding sites of the docking model for (*R*)-2-aminoheptane, (*R*)-1-phenylethylamine, ethylpyruvate and methoxyacetone, respectively. The large binding pockets and small binding pockets are shown as pink spheres and cyan spheres, respectively. The four ligands are shown as white sticks while the internal aldimine composed of PLP and Lys188 is shown as green sticks.

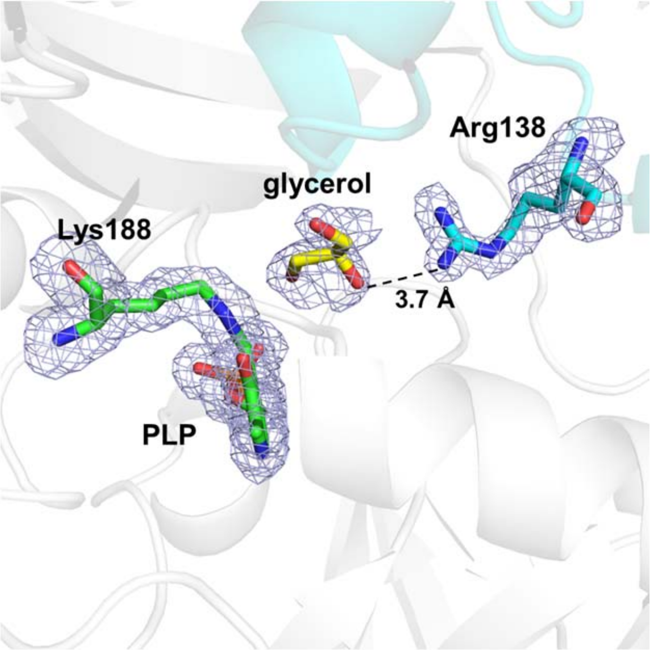

Supplementary Figure 5. View of the glycerol molecule between the two protomers of Ab-*R*-ATA. The internal aldimine formed by Lys188 and PLP, the glycerol molecule and the residue Arg138 from the adjacent protomer are shown as green, yellow and cyan sticks, respectively. The electrostatic interaction between glycerol and Arg138 is shown by a black dashed line. The F_o-F_c omit map for the internal aldimine, glycerol and Arg138, contoured at 2.5 σ, is shown as light blue mesh.

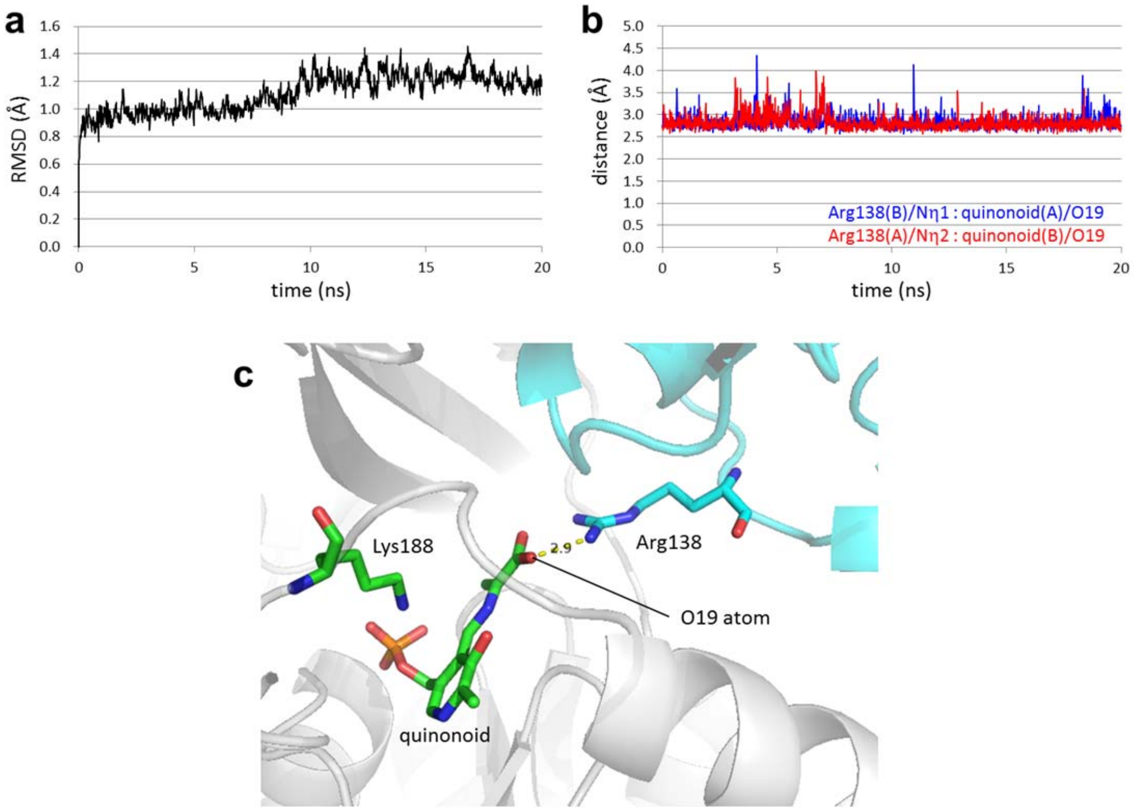

Supplementary Figure 6 Molecular dynamics simulation of Ab-*R*-ATA complexed with PMP-pyruvate quinonoid intermediate. (**a**) The RMS deviations of Cα atoms. The overall structure of the complex is stable after 0.2 ns. (**b**) Interatomic distances between Arg138 and the PMP-pyruvate quinonoid intermediate during the molecular dynamics simulation. The distance between Nη atom of Arg138 of chain B and the carboxyl oxygen atom of the quinonoid in the active site of chain A is shown in blue, while the other pair of Arg138 and the quinonoid is shown in red. (**c**) A snapshot of the interaction between Arg138 and PMP-pyruvate quinonoid intermediate at 6.5 ns.

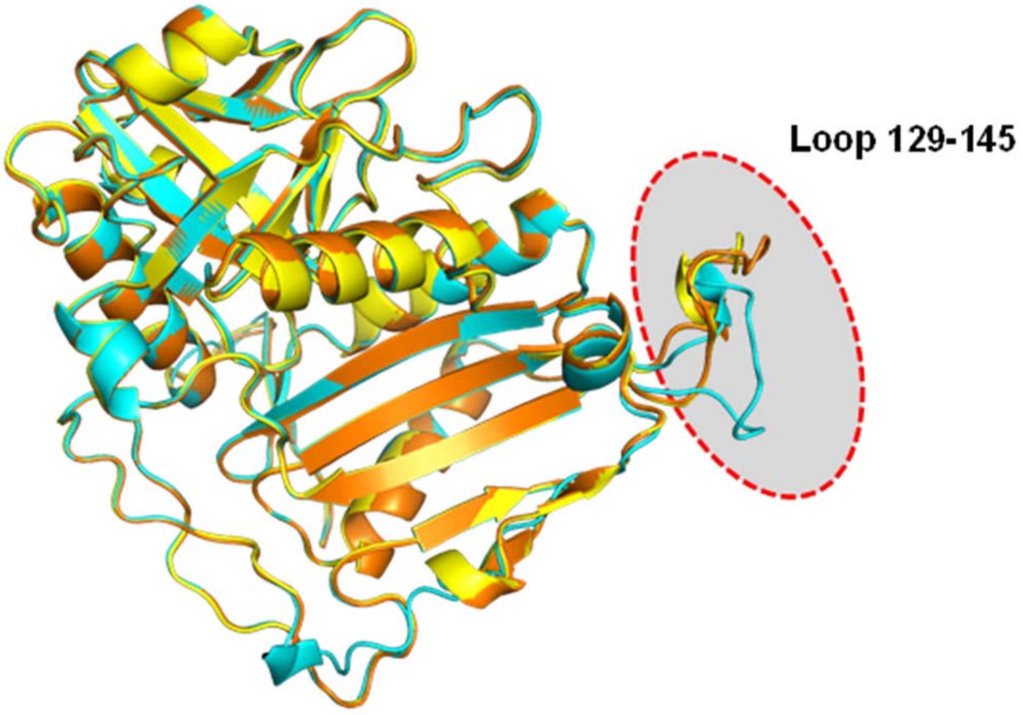

Supplementary Figure 7. Overall structure alignment of Ab-*R*-ATA, the G136F mutant and ATA-117-Rd11. The protomers of Ab-*R*-ATA, the G136F mutant and ATA-117-Rd11 are shown in a cartoon representation in cyan, orange and yellow, respectively. The three crystal structures are almost the same except for the loop 129-145, which is surrounded by a red dashed oval.

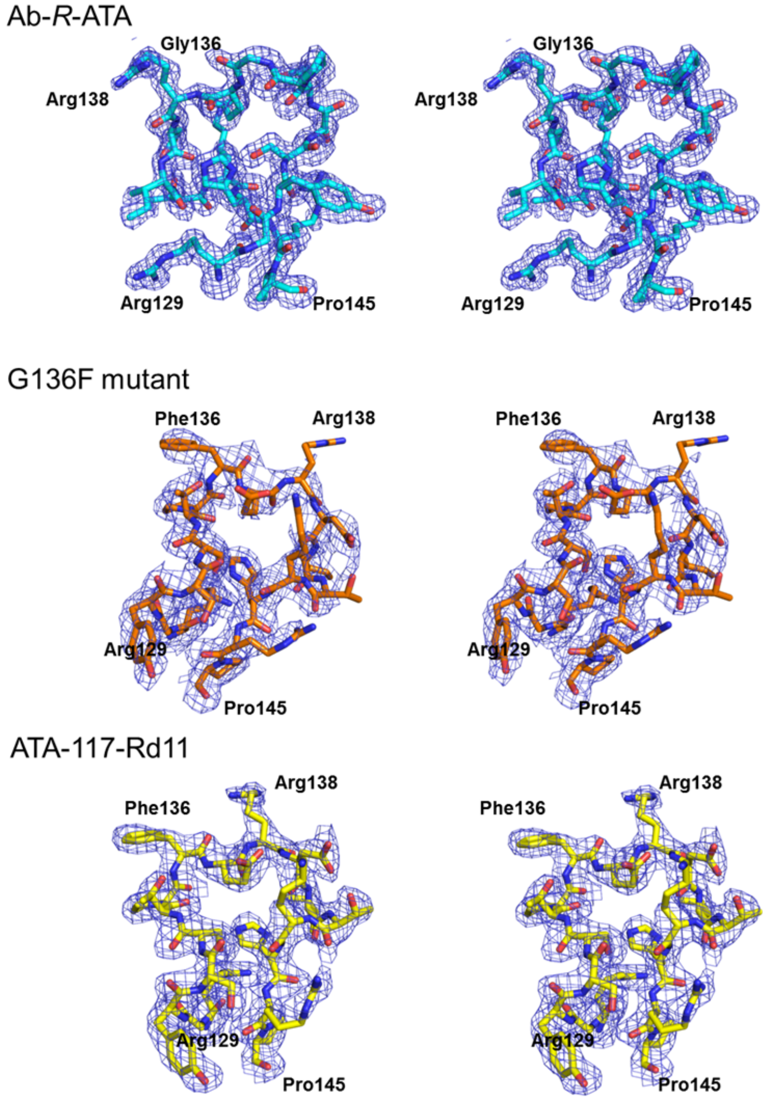

Supplementary Figure 8. F_o-F_c omit map for the loop 129-145 for Ab-*R*-ATA, the G136F mutant and ATA-117-Rd11. The maps are shown as light blue mesh contoured at 2.5 σ.

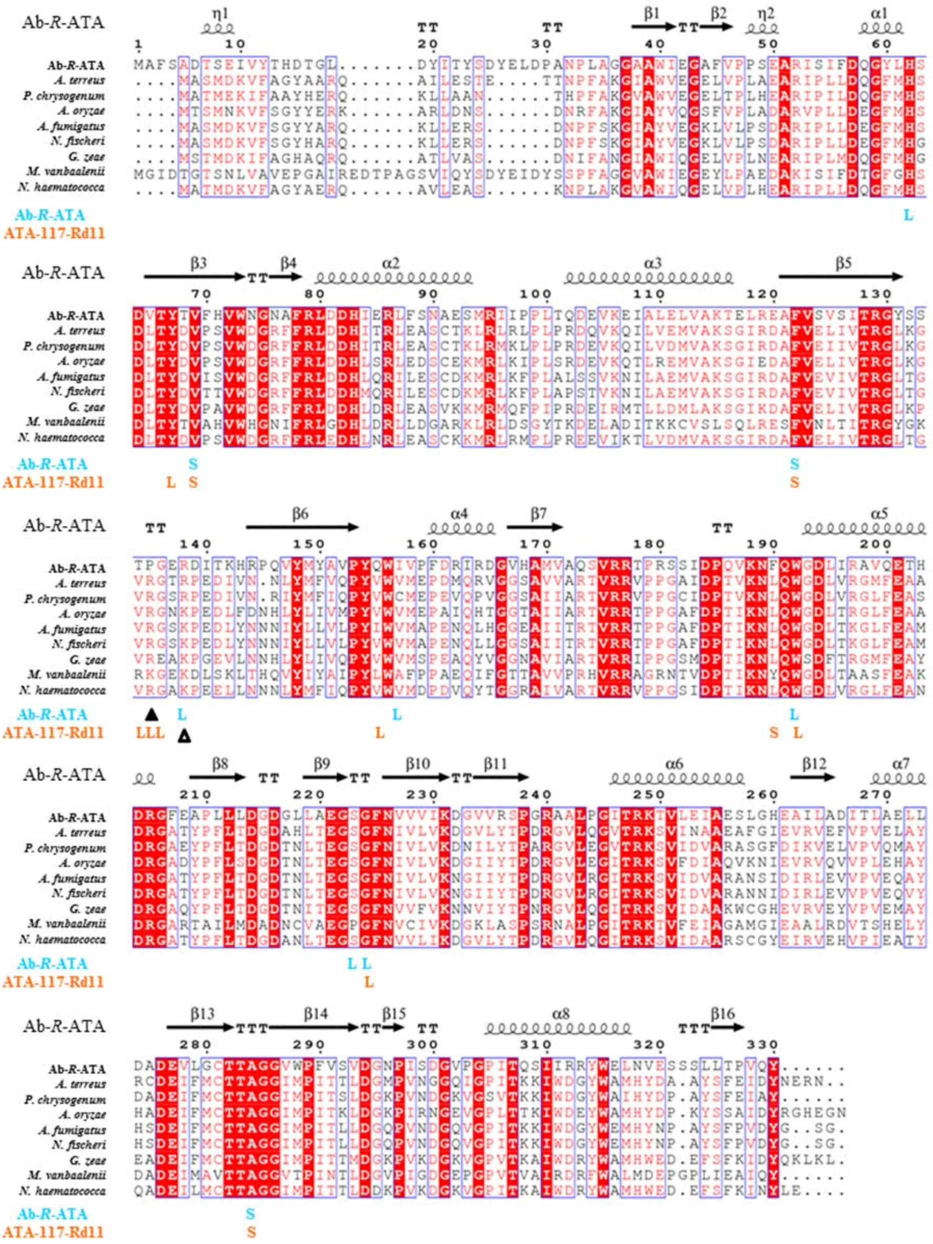

Supplementary Figure 9. Sequence alignment of nine *R*-ATAs. Strictly conserved residues are boxed in white on a red background and highly conserved residues are boxed

in red on a white background. At the top of the sequences, schematic representations of the secondary structure elements of Ab-*R*-ATA are shown. The α helix is depicted by a coil and the β strand is depicted by an arrow. Alignment was generated by ClustalW and the figure was generated by ESPript. The residues in the small binding pocket and large binding pocket of Ab-*R*-ATA and ATA-117-Rd11 are labeled with cyan characters and orange characters, respectively. The loops 129-145 are highlighted in a green box. Arg126 of the *R*-ATA from *Aspergillus fumigatus* and Arg128 of the *R*-ATA from *Aspergillus terreus* are indicated by filled black triangle. Arg138 of Ab-*R*-ATA is indicated by open black triangle. The homologs of Ab-*R*-ATA are from *Aspergillus terreus* (GI_115385557), *Penicillium chrysogenum* (GI_211591081), *Aspergillus oryzae* (GI_169768191), *Aspergillus fumigatus* (GI_70986662), *Neosartorya fischeri* (GI_119483224), *Gibberella zeae* (GI_46109768), *Mycobacterium vanbaalenii* (GI_120405468) and *Nectria haematococca* (GI_597960025).

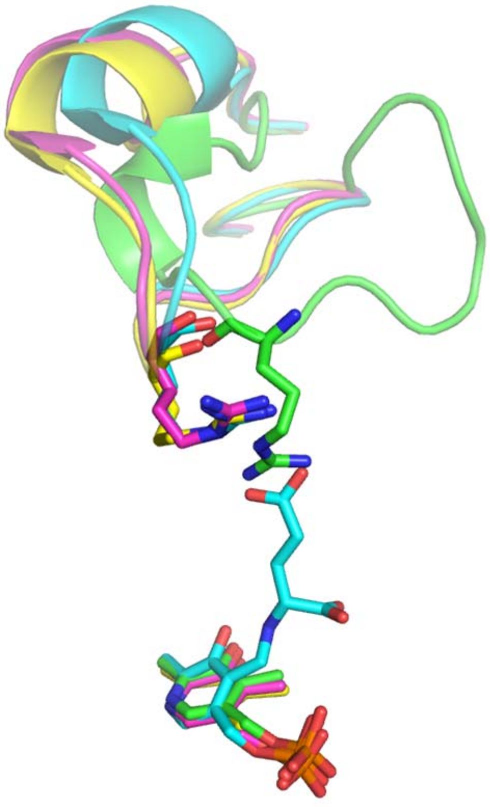

Supplementary Figure 10. The comparison of the Arg residues pointed into the active site. Arg138 of Ab-*R*-ATA, Arg128 of *R*-ATA from *Aspergillus terreus* (PDB ID 4CE5), Arg126 of *R*-ATA from *Aspergillus fumigatus* (PDB ID 4CHI) and Arg126 of *R*-ATA from *Nectria haematococca* (PDB ID 4CMD) are shown in green, cyan, magenta, and yellow sticks, respectively. The external aldimine and PLP moieties of internal aldimine are also shown in the same representation.

Supplementary Table 1. Data collection and refinement statistics

	**Ab-*R*-ATA**	**ATA-117-Rd11**	**Ab-*R*-ATA G136F**
**Data collection**			
Beamline	PF BL5A	PF AR-NW12A	PF BL5A
Wavelength (Å)	1.0000	1.0428	1.0000
Space group	$P\ 4_22_12$	$P\ 2_1$	$P\ 2_1$
Cell dimensions (Å)	80.62, 80.62, 93.88	82.01, 133.54, 195.54	81.96, 134.28, 196.04
Cell angles (Å)	90.00, 90.00, 90.00	90.00, 100.41, 90.00	90.00, 100.35, 90.00
Resolution (Å)	45.0-1.65	20.0-2.20	50.0-2.27
Number of observations	485000	719999	725546
Number of unique reflections	37724	201959	190856
Completeness (%)	99.9 (98.4)	96.3 (90.2)	98.8 (92.2)
R_{merge} (%)	6.1 (70.1)	6.2 (28.6)	8.5 (65.5)
Redundancy	12.86	3.57	3.80
$I/\sigma\ (I)$	31.93 (2.53)	13.99 (3.83)	11.6(1.99)
**Refinement**			
Resolution range (Å)	40.3-1.65	20.0-2.20	40.0-2.27
R_{work}/R_{free} (%)	15.4/17.2	16.8/21.3	17.1/21.8
Number of non-hydrogen atoms			
Protein	2566	30165	30292
Water	324	1802	1856
Ligand	PLP, glycerol	PLP	PLP
	21	192	180
RMSD bond length (Å)	0.007	0.009	0.012
RMSD bond angle (°)	1.209	1.252	1.455
Ramachandran plot (%)			
Preferred	97.6	97.1	97.8
Allowed	2.4	2.8	2.1
Outliers	0.0	0.1	0.1

The values in parentheses are for the highest resolution shell.

$R_{work}=\Sigma_{hkl}||F_o|-|F_c|| / \Sigma|F_o|$, where F_o is the observed structure factor and F_c is the calculated structure factor. R_{free} was calculated with 5% of reflections omitted from the refinement.

Supplementary Table 2. Kinetic parameters of Ab-*R*-ATA and its mutants

	Specific activity for pyruvate[b] U[a]/mg	Specific activity for benzylacetone[b] U[a]/mg	K_m for pyruvate[b] (mM)	k_{cat} for pyruvate[b] (s^{-1})	k_{cat}/K_m[b] ($s^{-1}mM^{-1}$)
WT	11.3±0.2	0.33±0.01	5.8±0.1	14±0	(240±10)×10^{-2}
R138Q	0.85±0.09	0.08±0.00	66±3	3.4±0.1	(5.2±0.1)×10^{-2}
R138A	0.86±0.02	0.16±0.00	110±20	9.2±1.1	(8.1±0.3)×10^{-2}
G136Y	0.47±0.01	0.38±0.01	350±20	12±0	(3.3±0.1)×10^{-2}
G136F	0.21±0.01	0.94±0.11	1300±100	15±1	(1.2±0.0)×10^{-2}
G136H	1.11±0.01	0.40±0.01	120±10	11±0	(8.8±0.4)×10^{-2}
G136W	0.65±0.04	0.18±0.01	580±90	23±2	(4.1±0.3)×10^{-2}

(a) One unit of Ab-*R*-ATA and mutants was defined as the amount of enzyme producing 1 μmol of D-alanine or (*R*)-amine per minute.

(b) The values are given as mean ± SEM of three independent experiments.
